# Bimaxillary orthognathic surgery with mentoplasty and the use of porous polyethylene

**DOI:** 10.1590/0103-644020266678

**Published:** 2026-05-01

**Authors:** Maria Clara Furlaneto Heck, Thales Fabro Vanzela Sverzut, Alexandre Elias Trivellato, Cássio Edvard Sverzut

**Affiliations:** 1 Department of Oral and Maxillofacial Surgery and Trauma and Periodontics - School of Dentistry of Ribeirão Preto of the University of São Paulo, Avenida Do Café, s/n, Monte Alegre, Ribeirão Preto, São Paulo14040‑904, Brazil

**Keywords:** dento-skeletal deformity, orthognathic surgery, porous polyethylene, multiprofessional

## Abstract

A clinical case of a patient with class III dento-skeletal deformity accompanied by deficient zygomatic prominence. The treatment plan included bimaxillary orthognathic surgery with the placement of porous polyethylene based biomaterial and mentoplasty. In the postoperative period, physiotherapeutic tapes were implemented to control swelling and prevent bruising. The importance of emotional and mental support for a successful recovery is emphasized, as is the need for careful planning and a multidisciplinary approach to ensure the best possible outcome. Trust between surgeon and patient, along with an understanding of the complexity of rehabilitating not only their occlusion, but also their self-esteem, are crucial aspects to consider.

## Resumo

Abordagem de caso clínico de uma paciente com deformidade dento-esquelética do tipo Classe III, acompanhada de deficiência de proeminência zigomática. A Cirurgia Ortognática bimaxilar, com a colocação de biomaterial à base de polietileno poroso e mentoplastia compuseram o plano de tratamento. No pós-operatório, o uso de faixas fisioterápicas foi implantado para o controle do inchaço e prevenção de hematomas. A importância do apoio emocional e mental foi destacada para uma recuperação bem-sucedida, além da necessidade de planejamento cuidadoso e uma abordagem multiprofissional para garantir o melhor resultado possível. A confiança entre cirurgião e paciente, juntamente com a compreensão da complexidade não apenas da reabilitação de sua oclusão, mas também da sua autoestima, são aspectos cruciais a se considerar.

**Figure:**
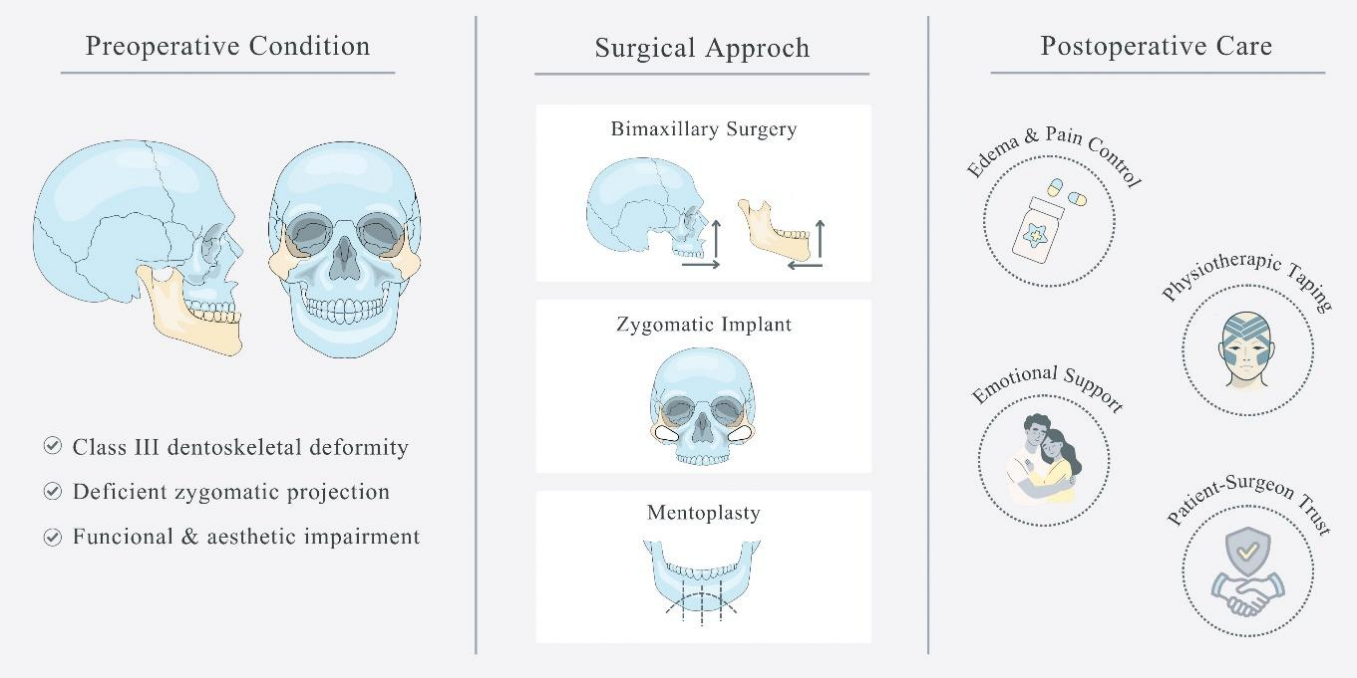

## Introduction

Dento-skeletal deformities represent abnormal facial proportions or an irregular relationship between the dental arches, ^1^ which can be treated with orthognathic surgery, a procedure that repositions the upper and/or lower jaws, to achieve greater harmony between the facial thirds. ^2^

Class III skeletal malocclusion is characterized by a maxillomandibular discrepancy in which the lower third is significantly larger than the middle third in the anteroposterior direction. ^3^ In certain cases, the anteroposterior growth of the middle third is deficient, resulting in maxillary retrusion together with a lack of zygomatic prominence ^4^ ^,^ ^5^. These aspects can be treated by combining orthognathic surgery and the use of alloplastic materials, such as porous polyethylene (Medpore™️, Stryker Craniomaxillofacial, Kalamazoo, MI, EUA), which promotes contour modification and increase zygomatic projection. ^6^

This report describes the simultaneous use of bimaxillary orthognathic surgery, mentoplasty, and porous polyethylene implants guided by a 3D-printed prototype, emphasizing both functional and aesthetic rehabilitation. Even though patient specific implants are available, pre-molded porous polyethylene implants still remain a good and cost-effective alternative. Furthermore, physiotherapeutic taping was applied as a postoperative management strategy to control swelling.

## Case report

**Figure 1:**
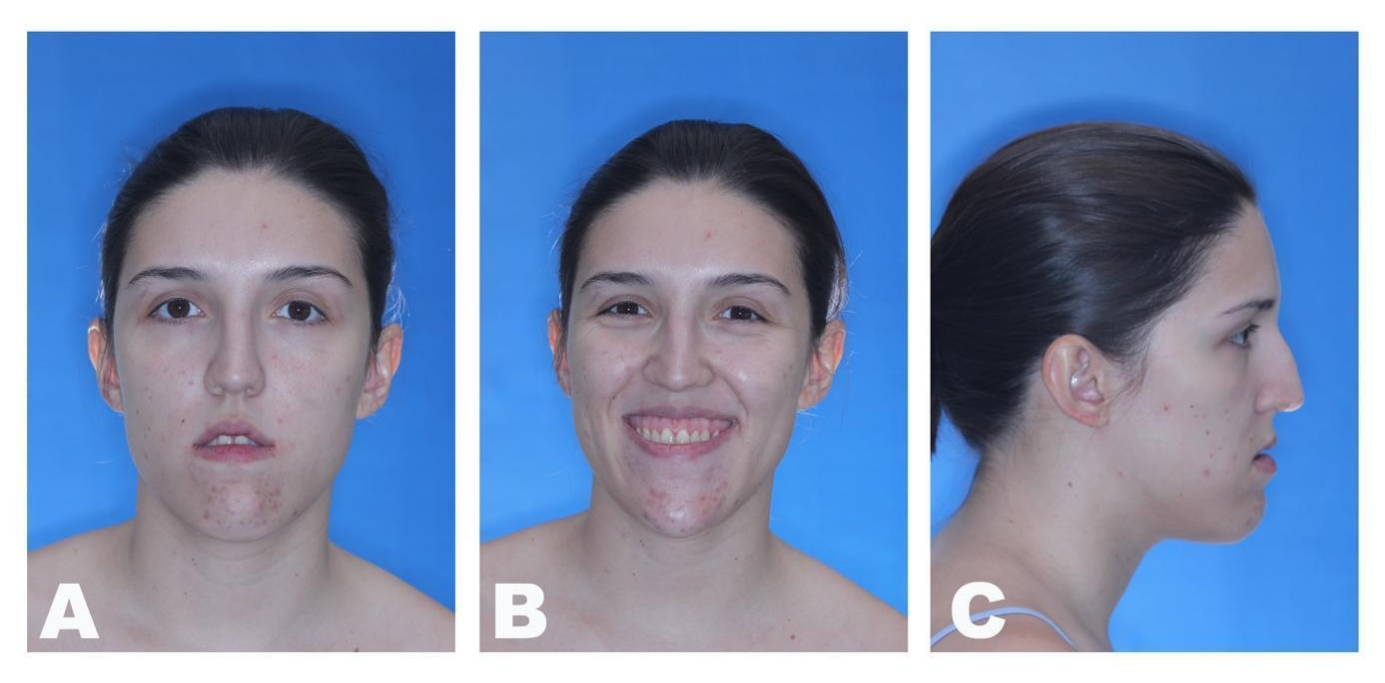
Preoperative facial images: A) Frontal view showing maxillary vertical excess resulting in lip incompetence; B) Frontal smiling view showing a remarkable gummy smile; C) Lateral view showing the deficiency of the zygomatic bone prominence and increased chin projection.

**Figure 2:**
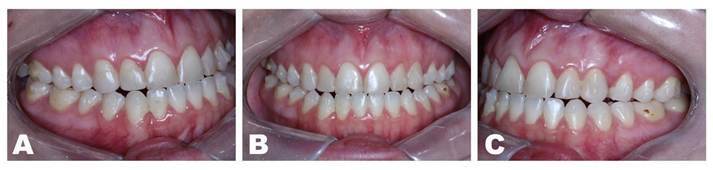
Preoperative dental images: A) Right side showing the canine and first molar class III relation; B) frontal view showing the anterior teeth touching top to top and the misalignment of the dental midlines; C) Left side showing the canine and first molar class III relation.

A 22 year-old female patient with normal oral and systemic health presented mandibular protrusion and a gummy smile as main complaints, highlighting aesthetic impairment (Figures 1 and 2). After thorough anamnesis, clinical examination and cone beam computed tomography scan evaluation, class III skeletal malocclusion was diagnosed, accompanied by deficient development of the zygomatic bones and increased chin projection.

### Treatment plan and preoperative

The proposed treatment plan was to perform bimaxillary orthognathic surgery, given the significant facial discrepancy and lower chance of relapse, together with the placement of porous polyethylene-based zygomatic implantes and mentoplasty.

Porous polyethylene was the chosen biomaterial, considering its advantages, disadvantages and previous surgical experience. It allows for easy fixation and adaptation, while presenting high biocompatibility, dimensional stability and low morbidity, allowing vascular and soft tissue growth within 1 week and bone tissue growth within 3 weeks. ^7^ ^,^ ^8^ However, it cannot be placed in areas subjected to mechanical pressure and the risk of infection must be considered ^9^ ^,^ ^10^.

The patient's preparation for the surgical procedure was based on the orthodontist’s work, which consisted of dental alignment, leveling and decompensation through the use of dental aligners, over a period of 8 months. Once the preoperative orthodontic mechanics had been completed, a conventional orthodontic appliance with a passive rectangular wire was placed for 3 weeks, allowing for precise intra-oral scanning and effective intraoperative maxillomandibular fixation. The surgical planning was entirely computer-assisted. The use of digital planning software reduces clinical time and improves the predictability of the surgical guide. ^11^ ^,^ ^12^ ^,^ ^13^

In addition, the patient was under professional psychological care, which played a crucial role in emotional and mental preparation, contributing to a better prognosis, as well as greater satisfaction and well-being. ^14^ ^,^ ^15^

### Surgery

**Figure 3:**
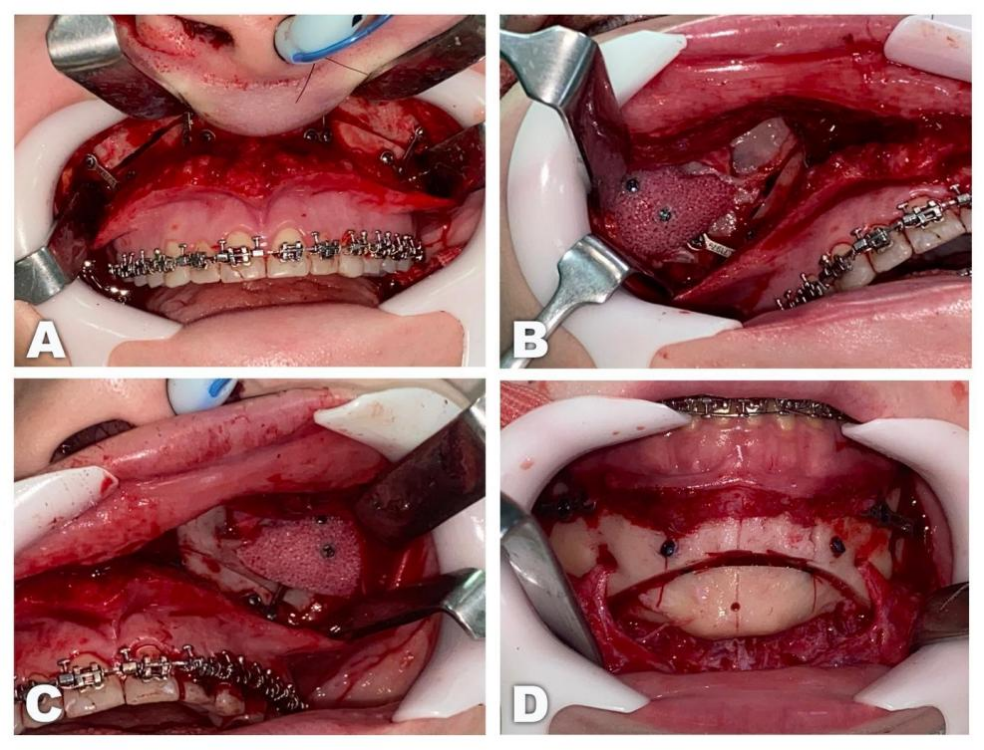
Intraoperative images: A) The Le Fort I osteotomy fixed with 1.5mm fixation system; B) The alloplastic biomaterial on the right side fixed with two 1.5mm screws; C) The alloplastic biomaterial on the left side fixed with two 1.5mm screws; D) Mentoplasty fixed with two positional screws from the 2.0mm system.

The bimaxillary orthognathic surgery performed consisted of Le Fort I osteotomy as proposed by Trimble et al. ^16^, and the bilateral sagittal split osteotomy as proposed by Epker ^17^. The maxilla was repositioned to 4mm anteriorly and 5mm superiorly. Fixation was then carried out by applying four 1.5mm “L” shaped plates positioned bilaterally on the canine and zygomatic buttresses and four 5mm length screws in each. (Figure 3A). Afterwards, applying the final guide obtained from the treatment plan, the mandible was advanced 0.02mm, superiorly repositioned by 5.84mm and the midline was repositioned 2.16mm to the right. The segments were fixed with one 2.0mm plate and four 5mm length screws on each side.

**Figure 4:**
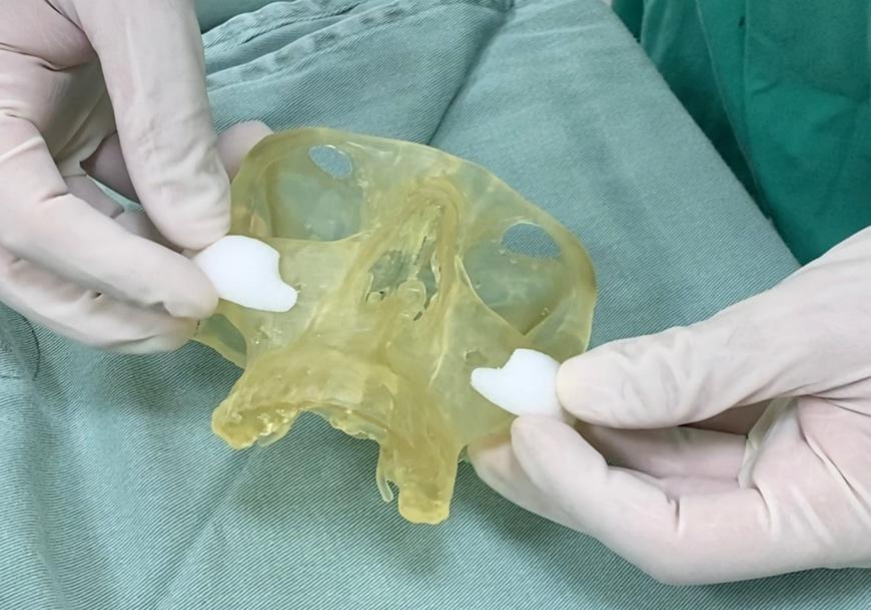
The adaptation of the porous polyethylene biomaterial on the 3D printed prototype after being immersed in hot saline water, making it malleable.

Furthermore, the adaptation of the porous polyethylene biomaterial was guided by a 3D printed prototype of the patient's upper and middle thirds. Immersing the alloplastic material in hot saline water made it malleable and allowed it to be positioned more precisely on the prototype (Figure 4), which led to less manipulation of soft tissues during its placement and stabilization with two 1.5mm screws with 10.0mm in length, resulting in improved zygomatic projection (Figures 3B and 3C). Chin prominence was altered using the mentoplasty technique, which consisted of a 4mm reduction and a 3mm setback, two 2.0mm positional screws with lengths of 14mm were used for fixation (Figure 3D), thus, chin projection and height were reduced, providing better facial aesthetics and harmony between patient's facial thirds.

### Postoperative

**Figure 5:**
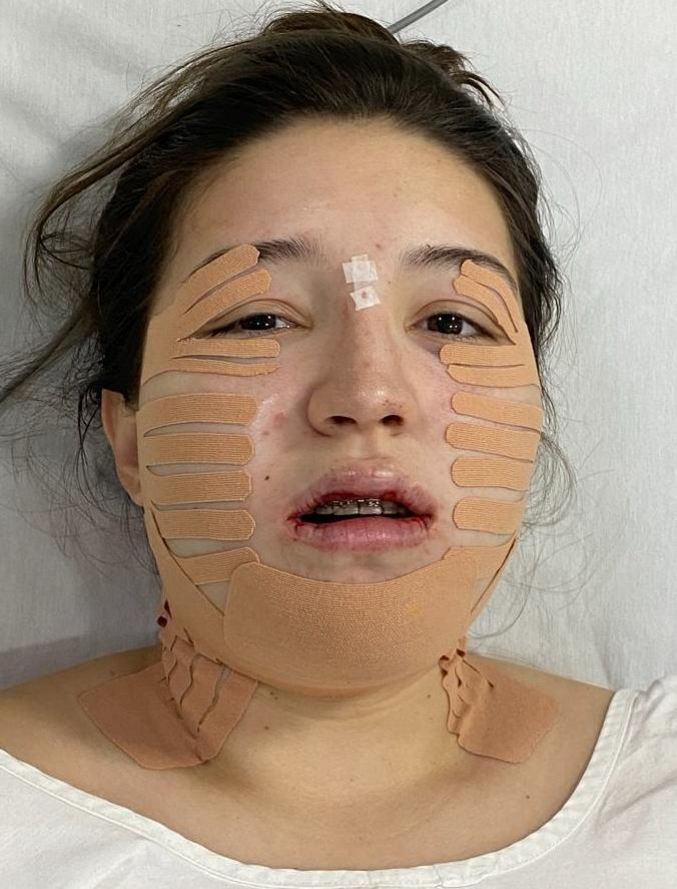
Patient in the recovery room with physiotherapy tapes strategically positioned, aiming to control edema and prevent hematomas.

In the recovery room, the patient was fitted with physiotherapy tape by a trained professional, strategically positioned to control edema and prevent hematoma, consequently optomizing treatment results. ^18^ ^,^ ^19^ Physiotherapy sessions were also carried out during her recovery process. (Figure 5)

The main post-surgical recommendations were: adequate rest, especially during the first few days after surgery, liquid food for 4 weeks, correct use of the prescribed medications to control pain, edema and infection, as well as attending follow-up appointments faithfully and keeping the responsible team aware of any abnormalities.

**Figure 6:**
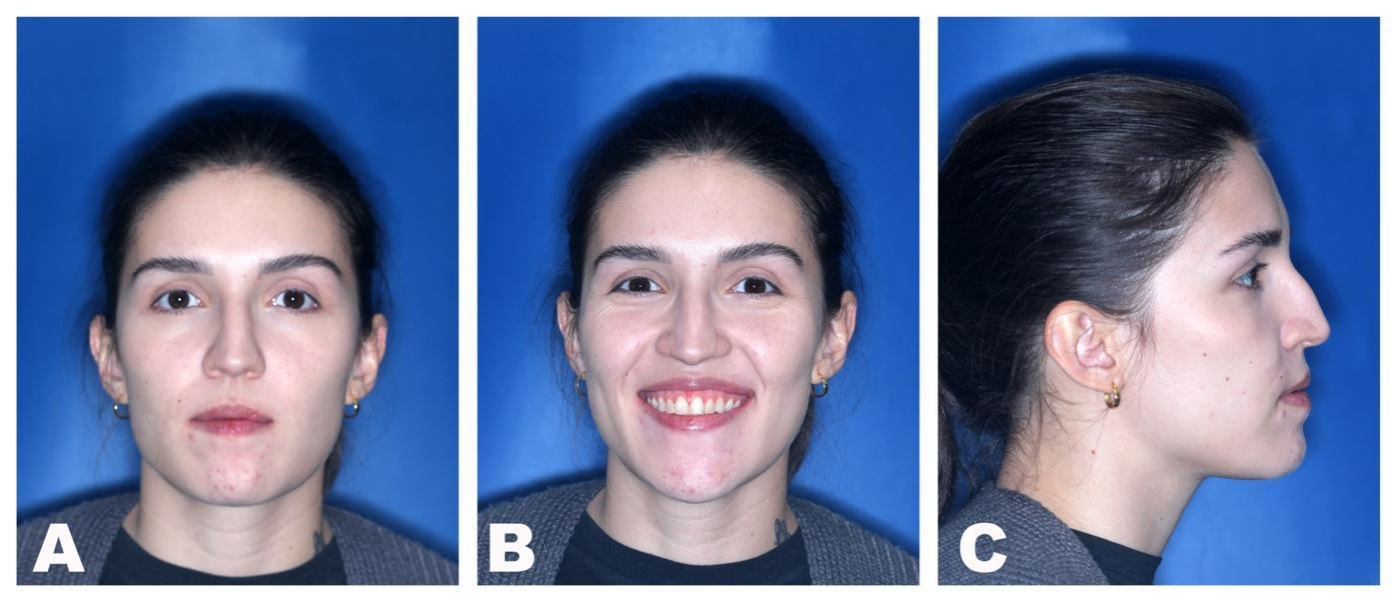
Postoperative facial images 22 months after the surgery: A) Frontal view showing an adequate balance between the facial thirds and the passive lips closure; B) Frontal smiling view showing an adequate gum exposure; C) Lateral view showing adequate zygomatic bone prominence and chin projection.

**Figure 7:**
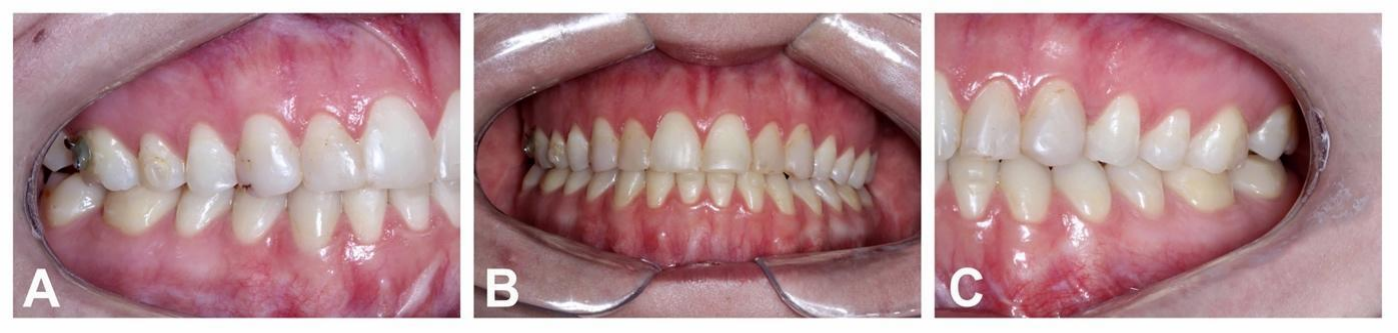
Postoperative dental images 22 months after the surgery: A) Right side showing the canine and first molar class I relation; B) frontal view showing an adequate overbite and relation between the dental arches and a misalignment of the dental midlines; C) Left side showing the canine and first molar class I relation.

**Figure 8:**
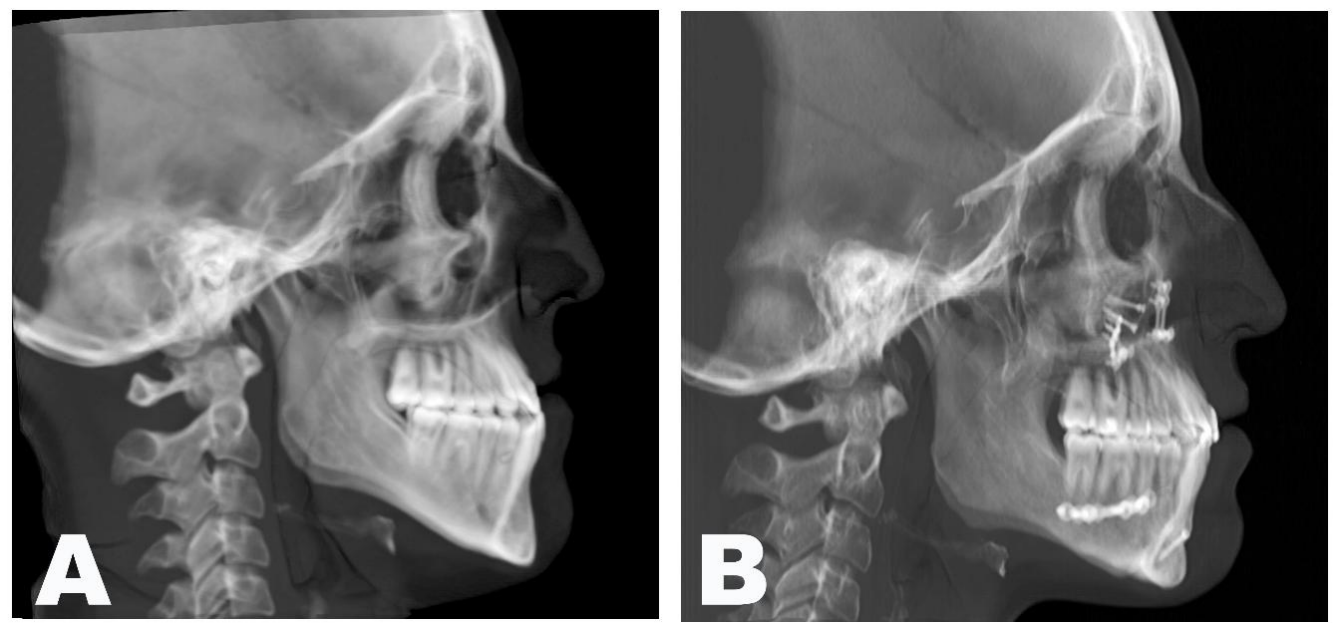
Lateral cephalometric radiograph: A) Preoperative and, B) Twenty-two months after the surgical procedure.

At the twenty-two months of follow-up, the patient showed satisfactory facial balance and occlusion (Figures 6 and 7), demonstrating maintenance of skeletal stability and implant integration, with no clinical or radiographic evidence of infection, resorption, or material displacement (Figure 8). Facial contour and symmetry remained stable, reinforcing the predictability and biocompatibility of the chosen biomaterial. (Figure 7).

However, misalignment between the dental midlines was still present at this time frame due to improper use of the aligners by the patient. The orthodontist was aware of and managing the issue (Figure 7B). Furthermore, the radiographs showed adequate repositioning and stability of the bone fragments (Figure 8).

It is worth emphasizing the importance of emotional and mental support, given the great difficulties faced, especially in the early stages of the post-operative period, to achieve a successful recovery from both a surgical and personal point of view for the patient. ^20^ ^,^ ^21^ ^,^ ^22^

## Discussion

Facial implants are indicated in cases of deficient volume or asymmetry, aiming to aesthetically reestablish facial proportions. Most devices are alloplastic, commonly manufactured from titanium mesh or high-density porous polyethylene, the latter facilitates osseous and fibrous ingrowth and therefore enhances long-term stability. Souza et al. ^22^ report that this material exhibits favorable biocompatibility and predictable clinical behavior, reinforcing its suitability for facial reconstruction. Kauke-Navarro et al. ^23^ compared different biomaterials used in facial reconstruction and reported that high-density porous polyethylene shows a relatively low infection rate among alloplastic options. The authors also highlighted that patient-specific implant design (PSI) significantly improves stability and reduces complication rates, reinforcing the importance of individualized three-dimensional planning in midface reconstruction. Although silicone is an alternative, we do not recommend the use this biomaterial, due to the absence of pores it becomes encapsulated, which facilitates removal in cases of infection, but promotes less stabilizationin the long-term. ^25^ ^,^ ^26^ ^,^ ^27^ ^,^ ^28^

Gerbino et al. ^29^, in a prospective study, analyzed three-dimensional bone and soft tissue changes after zygomatic valgization osteotomy associated with orthognathic surgery as proposed by Mommaerts et al. ^30^ and found it to be an effective and stable alternative for correcting zygomatic projection deficiencies. However, the additional osteotomy steps, including intentional fracture of the zygomatic body and the requirement for grafting material, substantially increase procedural morbidity. The authors further noted that a cranial osteotomy defect is always present and remains palpable without grafting, although not clinically visible.

Marella et al. ^31^ compared the functional results of titanium mesh and porous polyethylene in a clinical study, involving 8 patients, with post-operative follow-ups at different intervals. The results indicated that although both materials are effective, titanium mesh had a higher incidence of complications: infection and local sensitivity in the first few weeks after surgery. Conversely, the group treated with porous polyethylene showed statistically significant reduction in pain levels, suggesting better immediate post-operative comfort. These findings strengthen the preference for porous polyethylene, since early postoperative morbidity is a relevant clinical consideration.

In a comprehensive review, Rojas et al. ^32^ included clinical cases comparing the application of silicone and porous polyethylene. The incidence of infection and dislocation was higher in the silicone group, while the porous polyethylene group presented a higher occurrence of problems related to the prominence of the implant, especially in the malar region. Porous polyethylene implants are more common than silicone, and complication rates are low for both materials. The findings indicate that although both materials are safe, to optimize results, the choice should be guided by the anatomical region and the patient's risk profile.

In a prospective study, Niechajev ^33^ investigated the use of porous polyethylene implants, demonstrating effective tissue integration, with collagen formation and subsequent vascularization. Complications involved the need for adjustment or removal, however, 91% of cases remained stable over time. The material presents a low complication rate, morbidity levels similar to those associated with autogenous grafts, and high levels of patient satisfaction. Additionally, a review of orofacial aesthetic procedures highlighted the reliability of porous polyethylene for predictable and versatile soft-tissue volume augmentation. ^34^

A literature review by French et al. ^35^ evaluated 16 case series on porous polyethylene and PEEK zygomatic implants. Among 119 implants, only two (1.7%) required removal due to complications. The high success rate observed across this cohort suggests that malar implants represent a predictable and effective option for midface augmentation when applied according to established protocols. Recent evidence consolidates this perspective: Gugliotta et al. ^36^ reported sustained aesthetic and functional satisfaction in 37 patients reconstructed with patient-specific PEEK implants, with no displacement, fracture, or long-term infection during a mean 78.6-month follow-up.

The insertion of these materials in the zygomatic area must respect important anatomical structures, including: the infraorbital nerve, a branch of the maxillary nerve (V2), paresthesia or hypoesthesia may occur if injured; the zygomatic branch of the facial nerve (VII cranial nerve), which can result in facial asymmetry; the branches of the superficial temporal and maxillary arteries; and the facial vein, which can cause significant bruising or haemorrhage.^33^ The surgeon must pay attention to the zygomaticus major and minor muscles, the superior insertion of the masseter muscle and the orbicularis oculi muscle, as well as considering the proximity to the orbital cavity, whose lateral wall and floor are in close contact with the zygomatic bone, so inadvertent penetration can lead to diplopia, enophthalmos or serious eye damage.^2^ Such anatomical considerations reinforce the need for meticulous preoperative imaging and planning, and precise intraoperative navigation whenever available.

Despite favorable outcomes, porous polyethylene implants may present complications such as infection, late exposure, or displacement, particularly in pressure-bearing regions. Long-term stability depends on adequate fixation and vascularization. ^37^ ^,^ ^38^ Although our 22-month follow-up confirmed successful integration, extended surveillance remains crucial to validate durability in the long term.

Another limitation is inherent to material itself. Porous polyethylene, while biocompatible, lacks osteoconductivity and has lower mechanical strength than newer options such as PEEK or titanium. Its traditional, non-CAD/CAM fabrication also limits contour accuracy compared with patient-specific implants. Still, it remains a practical and cost-effective option when properly adapted and fixed intraoperatively.

The use of facial implants offers lower morbidity, greater safety and increased predictability, in addition to shorter postoperative recovery when compared to osteotomies, which are more invasive and complex procedures that modify the three-dimensional position of the zygoma to achieve anatomical correction. Thus, for patients requiring augmentation without skeletal repositioning, alloplastic implants represent a rational and more conservative alternative.

### Take-home message

This case underscores the effectiveness of integrating bimaxillary orthognathic surgery, mentoplasty, and porous polyethylene implants to correct Class III deformities associated with midface deficiency. Digital planning, combined with a multidisciplinary workflow, enabled precise surgical execution and achieved patient satisfaction.

Additionally, the incorporation of physiotherapeutic taping in the postoperative period served as a useful adjuvant therapy to minimize edema and improve patient comfort. Overall, the approach yielded stable aesthetic and functional outcomes over a 2-year follow-up.
